# Morbidity, Mortality, and Seasonality of Influenza Hospitalizations in Egypt, November 2007-November 2014

**DOI:** 10.1371/journal.pone.0161301

**Published:** 2016-09-08

**Authors:** Amr Kandeel, Patrick Dawson, Manal Labib, Mayar Said, Samir El-Refai, Amani El-Gohari, Maha Talaat

**Affiliations:** 1 Egypt Ministry of Health, Cairo, Egypt; 2 U.S. Naval Medical Research Unit No. 3, Cairo, Egypt; 3 Global Disease Detection Center, U.S. Centers for Disease Control and Prevention, Cairo, Egypt; National Institutes of Health, UNITED STATES

## Abstract

**Background:**

Influenza typically comprises a substantial portion of acute respiratory infections, a leading cause of mortality worldwide. However, influenza epidemiology data are lacking in Egypt. We describe seven years of Egypt’s influenza hospitalizations from a multi-site influenza surveillance system.

**Methods:**

Syndromic case definitions identified individuals with severe acute respiratory infection (SARI) admitted to eight hospitals in Egypt. Standardized demographic and clinical data were collected. Nasopharyngeal and oropharyngeal swabs were tested for influenza using real-time reverse transcription polymerase chain reaction and typed as influenza A or B, and influenza A specimens subtyped.

**Results:**

From November 2007–November 2014, 2,936/17,441 (17%) SARI cases were influenza-positive. Influenza-positive patients were more likely to be older, female, pregnant, and have chronic condition(s) (all p<0.05). Among them, 53 (2%) died, and death was associated with older age, five or more days from symptom onset to hospitalization, chronic condition(s), and influenza A (all p<0.05). An annual seasonal influenza pattern occurred from July–June. Each season, the proportion of the season’s influenza-positive cases peaked during November–May (19–41%).

**Conclusions:**

In Egypt, influenza causes considerable morbidity and mortality and influenza SARI hospitalization patterns mirror those of the Northern Hemisphere. Additional assessment of influenza epidemiology in Egypt may better guide disease control activities and vaccine policy.

## Introduction

Globally, acute respiratory infections (ARI) are the fourth leading cause of death [1] and second highest cause of years of life lost [2], and cause over 4.25 million deaths each year [3]. Previous studies have indicated that influenza comprises a substantial portion of ARI morbidity and mortality [4–5], with one estimate that 18% of global ARI deaths were due to influenza infection in 2010 [6] and another that influenza causes 250,000–500,000 deaths each year [7]. However, little is known about influenza epidemiology in many parts of the developing world, particularly in the Eastern Mediterranean Region (EMR).

The Arab Republic of Egypt, the second most populous country of the EMR, is a medium human-development country [8] located at the junction of northeastern Africa and southwestern Asia. Egypt has a desert climate with hot, arid summers and moderate winters. The population is over 86.8 million, and 32% are under 15 years old [9]. In Egypt, studies have evaluated limited aspects of human infection with avian influenza and the advent of the 2009 influenza pandemic [10–14]; however, nationwide data on influenza morbidity, mortality, and seasonality are limited.

Resolving knowledge gaps in influenza epidemiology in Egypt serves several purposes. First, a seasonal influenza vaccine is available each year that can prevent infections. Recent U.S. estimates of the effectiveness of Northern Hemisphere influenza vaccines ranged from 19–60% [15]. Even a moderately effective vaccine could reduce influenza-associated hospitalizations and lost worker productivity. Understanding whether influenza strains match those in either Northern or Southern Hemisphere circulation will elucidate whether Northern or Southern Hemisphere seasonal influenza vaccine is a viable policy. Additionally, data on whether demographic subpopulations are disproportionately affected by influenza may lead to designation of priority groups if vaccine availability is low. Second, improved understanding of the timing of influenza circulation may result in a more cost-efficient timing of resource allocation (e.g., antivirals) for hospitals and other clinical settings. This could reduce financial burdens on hospitals during times of elevated influenza transmission. Finally, enhanced data on annual influenza activity may provide a baseline metric to evaluate future activity. Therefore, influenza epidemics may be detected earlier allowing public health officials to respond faster and have more opportunity to interrupt transmission.

In 2007, the Eastern Mediterranean Acute Respiratory Infection Surveillance (EMARIS) Network was established to initiate sentinel surveillance for severe acute respiratory infections (SARI) to provide a better understanding of the epidemiology and viral etiologies of SARI in the EMR. The EMARIS Network was formed through a collaboration of participating countries’ ministries of health, U.S. Centers for Disease Control and Prevention (CDC), U.S. Naval Medical Research Unit No. 3 (NAMRU-3), and World Health Organization (WHO) Eastern Mediterranean Regional Office. We describe seven years of influenza epidemiology from the EMARIS-Egypt multi-site sentinel SARI surveillance system.

The aims of this study were to (1) assess the proportion of SARI cases having influenza infection in Egypt; (2) examine the types and subtypes of detected influenza viruses in Egypt; (3) compare demographic and clinical characteristics of influenza-positive SARI cases to those of influenza-negative SARI cases in Egypt; (4) quantify influenza deaths and assess influenza mortality risk factors in Egypt; and (5) establish a defined period of influenza seasonality in Egypt.

## Methods

### Setting and Study Design

SARI surveillance was initiated in Egypt in November 2007. Eight chest and infectious disease hospitals were selected based on their geographical distribution. All hospitals enrolled patients of all ages. The participating hospitals were Abbassia Chest Hospital and Abbassia Fever Hospital in Cairo, Alexandria Fever Hospital in Alexandria, Aswan Fever Hospital in Aswan, Damietta Chest Hospital in Damietta, Mahalla Fever Hospital in Gharbia, Minya Chest Hospital in Minya, and Shibin El-Kom Fever Hospital in Monufia.

A hospital surveillance team conducted SARI surveillance at each site. These teams included a surveillance coordinator, internist, pediatrician, and laboratory focal person, each of whom was trained on case definitions, enrollment procedures, specimen collection, and data recording. Teams screened all hospitalized patients with the SARI case definition. All hospitalized patients (including weekend admissions) who met the case definition and were admitted between November 1, 2007 and November 30, 2014 were eligible for enrollment, and those who provided informed consent were enrolled and assigned a unique study identification (ID) number.

### Definition of SARI Cases

The case definition for identifying eligible patients was standardized across the EMARIS Network. EMARIS participants reviewed the case definition each year, and it changed several times due to partner input and updated WHO guidance on SARI: from 2007–2009, the 2006 WHO SARI case definition was used [16]; from 2010–2011, the case definition became any hospitalized patient 31 days or older meeting the 2006 WHO definition, or any patient meeting the CDC International Emerging Infection Program pneumonia case definition [17], or any patient suspected to have SARI; from 2012–2014, the 2011 revised WHO SARI case definition was used (any hospitalized patient with a history of fever (≥38°C) and cough in the past seven days) [18], although clinicians could still enroll patients suspected to have SARI; and finally, in 2014, the symptom history was extended to the past ten days to align with the 2014 revised WHO SARI case definition [19].

### Sample Collection and Laboratory Procedures

Each enrolled patient had an oropharyngeal and nasopharyngeal swab taken with a sterile tip flocked with nylon fiber swab applicator within 24 hours of hospital admission. All collected swabs from a patient were placed in a 15 mL tube with 2 mL viral transport medium and agitated vigorously for 10 seconds in a vortex mixer. The resulting supernatant was split into two cryovials pre-labeled with the patient’s study ID number and stored in -70°C freezers before testing. Real-time reverse transcription polymerase chain reaction (rtRT-PCR) was conducted to detect influenza virus, types, and influenza A subtypes (determination of influenza B lineage was not done) [20]. Specimens were first tested at the hospital or Central Public Health Laboratory (CPHL) of the Egypt Ministry of Health (MOH), a recognized WHO National Influenza Center, and then at NAMRU-3, a WHO regional reference laboratory, for quality assurance. NAMRU-3 results were recorded as the gold standard final result by study ID into Microsoft Excel (Redmond, WA, USA). Laboratory results were recorded as influenza negative, A/H1N1, A/H1N1pdm09, A/H3N2, A/H5N1, A/unsubtyped, or B. For analytical purposes, specimens positive for two or more influenza types were coded according to the first specimen listed of: A/H5N1, A/H1N1pdm09, A/H3N2, A/H1N1, B, A/unsubtyped.

### Data Collection

Hospital surveillance teams completed a case report form including the unique study ID and demographic and clinical data for each enrolled patient. No other identifying information was recorded. The form was standardized across the EMARIS Network. Teams followed each patient prospectively until discharge, transfer to another hospital, or death, and collected data including age, sex, reported history of fever and cough, date of symptom onset, date of hospitalization, and date of last follow-up (i.e., the date of discharge, transfer to another hospital, or death). SARI cases and associated laboratory results were dated as the patient’s hospital admission date. Teams also abstracted data from medical records, such as chronic medical conditions and pregnancy status. Each form was entered into Microsoft Access at MOH and sent to a central database at NAMRU-3. Data managers performed double data entry and resolved any identified discrepancies amongst each other. The surveillance case report form database was merged with the laboratory result database, and the resulting database was cleaned and imported into SAS 9.4 (SAS Institute, Cary, NC, USA) for analysis.

### Statistical Analysis

Bivariate analyses of demographic and clinical data were conducted with the influenza laboratory result (positive versus negative) to generate counts and percentages and compare categorical levels by Pearson’s Chi squared test. The odds of death among influenza-positive cases were modeled with logistic regression using different explanatory variables (age group: pediatric <15 years old versus adult ≥15 years old; sex: male versus female; days from symptom onset to hospitalization: 0–2 versus 3–4 versus ≥5; chronic conditions: at least one versus none; and influenza type: A versus B). Statistical significance was set at an alpha level of 0.05. Continuous variables were assessed individually to generate the median, range, and interquartile range (IQR). Monthly influenza positivity rate was calculated as the proportion of specimens positive for influenza out of the total specimens tested for influenza each month.

Influenza seasonality was evaluated by examining the monthly average number and proportion of influenza-positive specimens. The influenza season was defined as beginning on the month having the lowest average influenza activity over the entire period and ending one year later. The month having the highest proportion of a season’s influenza-positive specimens was considered that season’s peak while the month having the lowest proportion was considered that season’s nadir.

### Ethical Considerations

NAMRU-3 Institutional Review Board (IRB), CDC Institutional Review Board, and Egypt MOH Institutional Review Board approved the SARI surveillance protocol in 2007 and maintained approval for the duration of the surveillance period. Written informed consent was not required by the IRBs due to the minimal risk faced by patients and because sentinel SARI surveillance is part of Egypt’s national routine respiratory infection surveillance system of the Egypt MOH (the requirement was waived), but verbal informed consent was required. Hospital surveillance teams provided consent information to patients both written and verbally in Arabic, allowing adequate time for consideration. Verbal informed consent was also obtained from the parents, caretakers, or guardians on behalf of all children/minors enrolled in the study. Verbal informed consent was documented by the enrolling physician signing the consent form if the patient approved. The informed consent process for this study was approved by the three aforementioned institutional ethical review boards.

## Results

### Influenza Morbidity in Egypt

From November 2007–November 2014, 17,441 patients met the SARI case definition and had a specimen tested for influenza by rtRT-PCR (S1 File). Of these, 2,936 (17%) were influenza-positive. A total of 2,013 (12%) were influenza A-positive and 923 (5%) were influenza B-positive. Of the 2,013 influenza A-positive specimens, 115 (6%) were subtyped as seasonal A/H1N1, 1,200 (60%) as A/H1N1pdm09, 674 (33%) as A/H3N2, 16 (1%) as A/H5N1, and 8 (<1%) as A/unsubtyped.

### Characteristics of Influenza SARI Cases in Egypt

Demographic and clinical characteristics of SARI patients by viral influenza result are shown in Table 1. Influenza-positive patients differed from influenza-negative patients by age group (p<0.0001). Among those with available data on age, 30% of influenza-negative patients were under five years old compared to 16% of influenza-positive patients. The median age of influenza-positive patients was higher than that of influenza-negative patients: 30 years (range 1 month–90 years, IQR 12–50 years) compared to 27 years (range 1 month–95 years, IQR 3–50 years). Including missing data, influenza-positive cases were more likely to be female (and if female, be pregnant), have a preexisting chronic condition, require ventilation, be admitted to an intensive care unit (ICU), and die (all p<0.0001). When missing data were excluded, statistically significant (p<0.05) differences persisted except for ventilation, ICU admission, and death.

**Table 1:** Characteristics of Patients Enrolled in Sentinel Surveillance for Severe Acute Respiratory Infection by Viral Influenza Result, Egypt, November 2007-November 2014.

	Influenza Positive	Influenza Negative	Missing Data	Non-Missing p-value†
Characteristic	(n = 2,936)	(n = 14,505)	(N = 17,441)	
	N (% Column Total)			
Age Group*			1,083 (06)	<0.0001
<2 Years	161 (05)	2,238 (15)		
2–4 Years	287 (10)	1,771 (12)		
5–14 Years	290 (10)	1,205 (08)		
15–49 Years	1,291 (44)	4,667 (32)		
50–64 Years	561 (19)	2,499 (17)		
≥65 Years	192 (07)	1,196 (08)		
Unknown	154 (05)	929 (06)		
Sex*			983 (06)	0.0005
Male	1,445 (49)	7,525 (52)		
Female	1,359 (46)	6,129 (42)		
Unknown	132 (05)	851 (06)		
Pregnancy Status^1^*			482 (17)^1^	<0.0001
Pregnant	128 (20)	255 (12)		
Not Pregnant	425 (67)	1,494 (17)		
Unknown	85 (13)	397 (19)		
Preexisting Conditions^2^*			3,873 (22)	0.0018
≥1 Chronic Conditions	803 (27)	3,145 (22)		
None	1,735 (59)	7,885 (54)		
Unknown	398 (14)	3,475 (24)		
Required Ventilation			3,762 (22)	0.0604
Yes	49 (02)	157 (01)		
No	2,512 (86)	10,961 (76)		
Unknown	375 (13)	3,387 (23)		
Intensive Care Unit			3,744 (21)	0.0749
Admitted	118 (04)	427 (03)		
Not admitted	2,448 (83)	10,704 (74)		
Unknown	370 (13)	3,374 (23)		
Outcome			4,397 (25)	0.5954
Death	53 (02)	226 (02)		
Discharge	2,268 (77)	10,497 (72)		
Unknown	615 (21)	3,782 (26)		

† Pearson’s Chi squared test (categories excluded missing data).

*p<0.05 (predetermined statistical significance level).

^1^Out of the total number of female individuals in the childbearing age range, 15–49 years old.

^2^Preexisting conditions include asthma, chronic obstructive pulmonary disease, tuberculosis, and other respiratory, cardiac, endocrine, hepatic, renal, neurologic, hematologic, or unspecified diseases.

The median duration from symptom onset to hospitalization among all patients was 3 days (IQR 2–4 among influenza-positive and 2–5 among influenza-negative), and this did not change by sex, age group, or both sex and age group. The median duration from hospitalization to end of follow-up among all patients was 5 days (IQR 4–6 among influenza-positive and 4–7 among influenza-negative), and this did not change by sex, age group, or both sex and age group.

### Influenza Mortality in Egypt

Of the 2,936 influenza-positive cases, 53 (2%) died. Influenza-positive deaths comprised 19% of SARI deaths. Among the 53 influenza deaths, 83% were 15–64 years old, 53% were male, and 55% had a preexisting chronic condition. Twelve percent (3/25) of females who had influenza and died were pregnant. Influenza A accounted for 83% of influenza-positive deaths, and of the 44 influenza A deaths, 70% were subtyped as A/H1N1pdm09, 18% as A/H5N1, 9% as A/H3N2, and 2% as A/unsubtyped. The SARI case fatality rates by influenza virus type were: 50% (8/16) A/H5N1, 13% (1/8) A/unsubtyped, 3% (31/1,200) A/H1N1pdm09, 1% (9/923) B, 1% (4/674) A/H3N2, and 0% (0/115) seasonal A/H1N1. The median duration from symptom onset to hospitalization and the median duration from hospitalization to end of follow-up were both 4 days among influenza-positive cases who died.

Factors associated with death among influenza-positive cases are displayed in Table 2. Adjusting for all covariates, the odds of death among influenza-positive cases were significantly greater for adult versus pediatric patients, patients having symptoms for five or more days before hospitalization versus two or less days, patients with a preexisting chronic condition, and patients having influenza A versus B (all p<0.05).

**Table 2:** Factors Associated with Death among Influenza-Positive Patients Enrolled in Sentinel Surveillance for Severe Acute Respiratory Infection, Egypt, November 2007-November 2014.

	Modeling Odds of Death	
Covariate	Crude OR (95% CI)	Adj. OR (95% CI)*
Age Group		
<15 Years (Pediatric)	Referent	
≥15 Years (Adult)	4.76 (1.71–13.24)	3.24 (1.14–9.24)
Sex		
Female	Referent	
Male	1.08 (0.63–1.87)	1.15 (0.65–2.03)
Symptom Onset to Hospitalization		
0–2 Days	Referent	
3–4 Days	1.50 (0.66–3.43)	1.61 (0.70–3.69)
≥5 Days	6.15 (2.74–13.76)	7.24 (3.17–16.57)
Preexisting Conditions^1^		
≥1 Chronic Conditions	3.01 (1.73–5.24)	2.56 (1.43–4.58)
None	Referent	
Influenza Virus		
Influenza A	2.32 (1.12–4.77)	2.96 (1.37–6.40)
Influenza B	Referent	

OR = Odds Ratio, CI = Confidence Interval, Adj. = Adjusted.

*Adjusted for all other covariates in table.

^1^Preexisting conditions include asthma, chronic obstructive pulmonary disease, tuberculosis, and other respiratory, cardiac, endocrine, hepatic, renal, neurologic, hematologic, or unspecified diseases.

### Influenza Seasonality in Egypt

**Fig 1:**
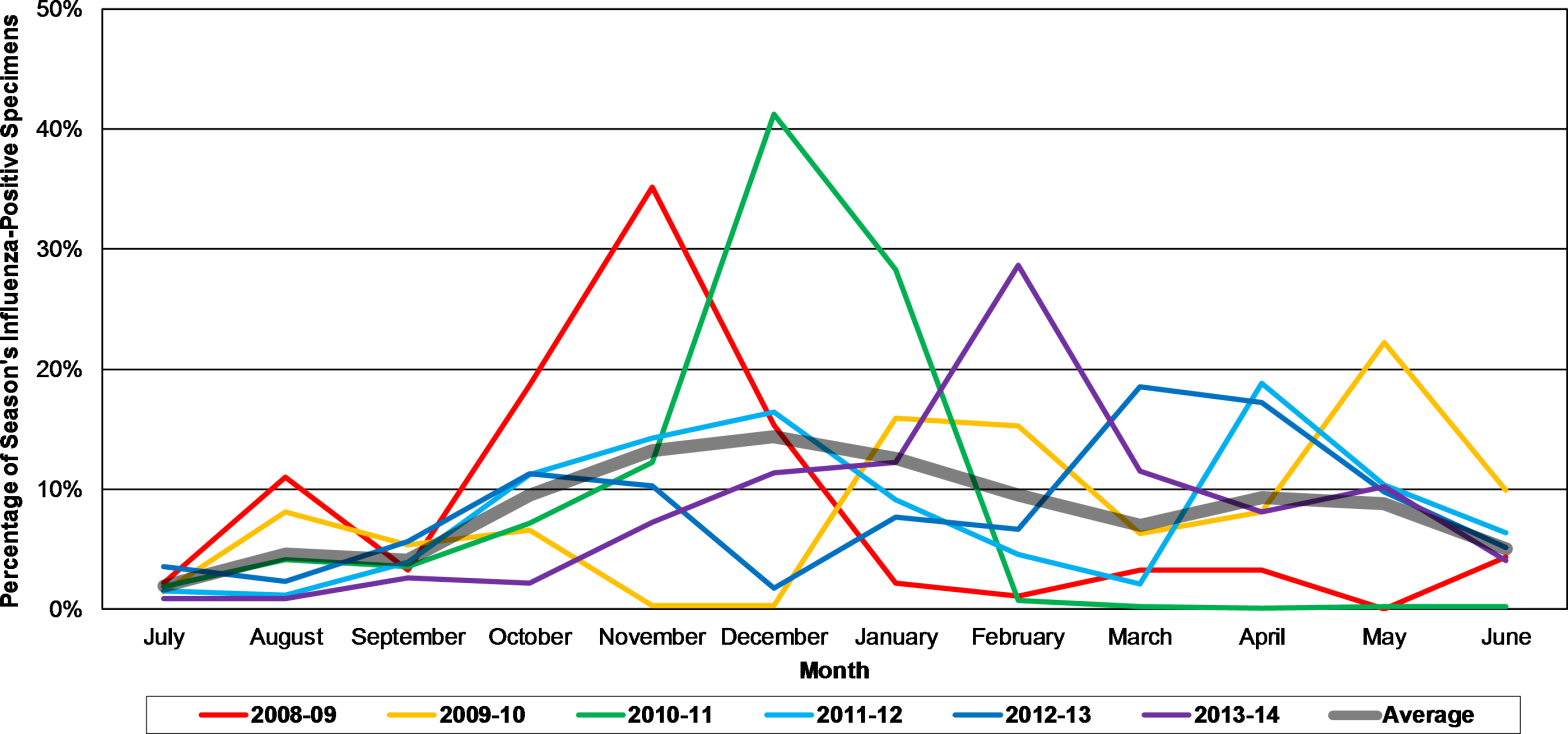
Percentage of Season’s Influenza-Positive Specimens by Month, Egypt, July 2008-June 2014. The percentage of each season’s influenza-positive specimens occurring in each month of the influenza season is displayed. Influenza seasons were defined as the one-year period from July to June.

The calendar month with the lowest average number and proportion of influenza-positive cases was July, making July the influenza activity nadir. The influenza season in Egypt was therefore defined as beginning in July and concluding in June. The calendar month having the highest average number and proportion of influenza-positive cases was December, making December the influenza activity peak. Seasonal influenza activity peaks (Fig 1) occurred in November (2008–09), December (2010–11), February (2013–14), March (2012–13), April (2011–12), and May (2009–10). In the months of peak activity, the month accounted for an average of 27% (range 19–41%) of the entire season’s influenza cases, and influenza accounted for an average of 37% of all SARI cases that month (range 30–50%).

**Fig 2:**
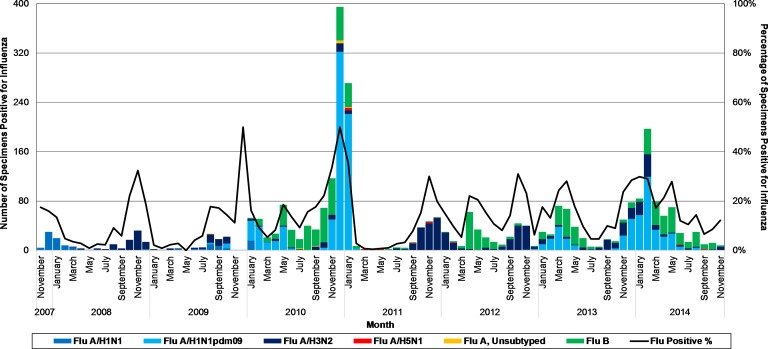
Number and Percentage of Severe Acute Respiratory Infection (SARI) Surveillance Patient Specimens Positive for Influenza by Viral Influenza Etiology and Month, Egypt, November 2007-November 2014 (n = 2,396). Vertical bars represent the raw number of influenza types and subtypes recorded each month (left vertical axis). The line represents the percentage of all patient specimens collected each month that were positive for any influenza type or subtype (right vertical axis).

The influenza virus types and subtypes detected over time are shown in Fig 2. In each season, the predominant influenza virus was A/H3N2 (2008–09 and 2011–12), A/H1N1pdm09 (2009–10, 2010–11, and 2013–14), or B (2012–13).

## Discussion

This is the first study to examine national influenza epidemiology and seasonality in Egypt. Data from Egypt’s SARI surveillance system were used to describe influenza hospitalizations at eight geographically-representative Egyptian hospitals across a seven-year period. We found that approximately one of every six SARI patients had an influenza infection, and during months of peak influenza activity, this was as high as one of every two. Influenza-positive patients were different from influenza-negative patients in a number of ways, though differences regarding ventilation, ICU admission, and death were inconclusive. Death among influenza-positive patients was associated with four characteristics. We also found marked influenza seasonality beginning in July each year and ending in June. Seasonal peaks occurred from November to May, and the peak month contained an average of 27% of the entire season’s influenza infections.

Our findings indicate that influenza leads to substantial morbidity and mortality in Egypt among people of all ages and both sexes. From 2007–2014, nearly 3,000 influenza hospitalizations were detected by the eight sentinel hospitals, approximately 17% of SARI hospitalizations. This figure is nearly double those recently found in other EMARIS Network countries: 8% in Oman and 9% in Jordan [21–22], and larger than estimates obtained in multiple African [5, 23] and Asian countries [24–25]. Furthermore, influenza accounted for 19% of SARI deaths in Egypt. This mirrors the recent worldwide estimate of 18% [6].

Comparison of demographic characteristics revealed that some individuals have increased odds of severe influenza infection in Egypt. Among the age groups encompassing 5–65 years, patients were more likely to have influenza. More influenza-negative patients were under five years old, possibly due to pediatric respiratory syncytial virus (RSV) infection which has been shown to be more prevalent among infants and young children in Egypt [13, 26–27]. A similar proportion of influenza-positive and influenza-negative patients were 65 years or older, possibly reflecting that elderly adults are simply predisposed to ARIs of various etiologies. Other factors associated with influenza among those hospitalized with SARI from this study include pregnancy and chronic conditions. Influenza-positive women were more likely to be pregnant, and numerous other studies have identified pregnancy as a risk factor for influenza and influenza complications [28–32]. Chronic conditions were also associated with influenza hospitalization, as 27% of influenza-positive patients had at least one. Previous studies have shown that chronic disease is a risk factor for influenza and complications due to influenza [33–36].

Although 2% of patients with influenza died, there was no apparent difference in mortality between influenza-positive and negative patients. Regardless, patients with influenza infection comprised 19% of total SARI deaths. Analysis of influenza mortality among hospitalized patients with influenza found four potential risk factors: older age, chronic conditions, a longer duration from symptom onset to hospitalization, and influenza A virus infection. Overall, the number of deaths was greater for those with influenza A than influenza B, which aligns with previous research [37–38]. The influenza subtype with the highest mortality (50%) was A/H5N1 avian influenza. As of March 3, 2015, the worldwide A/H5N1 case fatality rate was 55% [39], similar to our finding. Furthermore, though 13% of patients with influenza A/unsubtyped died, there were only eight such infections. Additionally, a longer duration from symptom onset to hospitalization increased the odds of death among influenza-positive patients. This supports previous research that found a longer duration from influenza symptom onset to hospitalization increased the odds of death [40–41], which may be due to individuals’ healthcare-seeking behavior, i.e., avoiding a hospital visit until it is too late for medical intervention.

In addition to influenza hospitalization morbidity and mortality in Egypt, we were able to assess seasonality over seven years. We observed an annual seasonal pattern beginning in July and ending the following June. Overall influenza activity peaked in November–May, with the peak month having 19–41% of that entire season’s influenza infections and accounting for 30–50% of all SARI cases that month. These seasonal peaks were largely driven by influenza A, and in some seasons, there were bimodal influenza peaks corresponding to an earlier influenza A peak followed by a later influenza B peak. Climate has been shown to correlate with influenza activity worldwide, particularly lower temperatures and lower relative humidity [42–44]. Although Egypt has a desert climate with year-round high temperatures [9], we observed annual Northern Hemisphere influenza seasonality with activity peaks in the winter and spring, perhaps due to lower temperatures during those months.

Based on these data, seasonal influenza vaccination in Egypt may be a useful recommendation. The system identified predominant influenza strains each season [45], indicating some seasonal influenza transmission could have been interrupted by vaccination. However, future studies are needed to antigenically characterize the predominant influenza strains in Egypt to assess their similarity to strains included in the corresponding season’s vaccine. Possible priority groups for vaccination in Egypt include pregnant women, individuals with chronic conditions, and older adults based on aforementioned findings.

Influenza seasonality in Egypt suggests an ideal timing for resource allocation and provides a baseline metric on which to compare future activity. These data indicate that Egyptian hospitals should prepare for peak influenza activity from November–May. Stockpiling antiviral medication, increasing staff capacity, and reserving hospital beds could begin in October and be scaled back in May. Also, MOH and partners could use these data to determine average weekly influenza hospitalization activity (smoothed with moving averages) for evaluation of future activity. This will allow for earlier discovery of unusual influenza activity, including crossing of epidemic thresholds, allowing for a faster public health intervention.

There were limitations to these data. First, the system’s detection of influenza relied on the case definition’s sensitivity and specificity, and the definition changed over time based on WHO guidance. It is possible the system missed some hospitalized individuals with influenza. However, the case definition was standardized across the network and the system detected a higher proportion of influenza SARI hospitalizations than other countries. Second, more comprehensive demographic and clinical data were unavailable, and thus more nuanced analyses of risk factors were not possible. This is a reality of surveillance as only basic data are collected to reduce staff burden. Third, data were missing for a sizeable number of individuals for some variables. Therefore, statistical comparisons were presented both including and excluding missing data, and some comparisons yielded divergent results (implying statistical significance was driven by the extent of missing data for some variables). Work to maintain the quality and integrity of surveillance data through the EMARIS Network is ongoing, including improving motivation among hospital surveillance teams.

Strengths of this study stem from its scale, length of observation, and capabilities to detect influenza virus. The use of eight sentinel hospitals representatively distributed throughout Egypt allowed for adequate generalizability of findings to the national level. Furthermore, with over 17,000 patients enrolled, there was ample power to detect meaningful differences in demographic, clinical, and virological characteristics. The seven-year period of surveillance afforded the opportunity to examine influenza seasonality over time, which is only possible with several years of data. Finally, Egypt’s CPHL and NAMRU-3 enabled sensitive and specific detection of influenza viruses and types via rtRT-PCR for all 17,441 SARI patients.

This is the first documented examination of national influenza epidemiology in Egypt. Given the dearth of influenza knowledge in the EMR, our study helps fill a critical epidemiologic gap for the second most populous country in the region. Our findings indicate that influenza is an important cause of morbidity and mortality. Furthermore, it appears to have Northern Hemisphere seasonality, though not as pronounced as in temperate climate countries. Therefore, seasonal influenza vaccination may be a viable policy. Ultimately, the EMARIS Network provided numerous benefits, namely increased epidemiologic and laboratory capacity, enhanced International Health Regulations (2005) compliance, and a sustainable SARI surveillance system. Such a system is a crucial component of modern disease control, as evidenced by the recent appearance of Middle East Respiratory Syndrome Coronavirus [46]. It may also be adapted for other novel ARIs, which is imperative given growing concern over the emergence of new influenza viruses with pandemic potential [47–48]. Continued surveillance for influenza and further assessment of its molecular epidemiology will better inform decision-makers about disease control activities in Egypt and the greater EMR.

## Supporting Information

S1 File Raw Data. Data file (SAS format) for the Severe Acute Respiratory Infection (SARI) Sentinel Surveillance System in Egypt.
(SAS7BDAT)
